# An Orchid in Retrograde: Climate-Driven Range Shift Patterns of *Ophrys helenae* in Greece

**DOI:** 10.3390/plants10030470

**Published:** 2021-03-02

**Authors:** Martha Charitonidou, Konstantinos Kougioumoutzis, John M. Halley

**Affiliations:** 1 Laboratory of Ecology, Department of Biological Applications and Technology, University of Ioannina, 45110 Ioannina, Greece; 2 Department of Ecology and Systematics, Faculty of Biology, National and Kapodistrian University of Athens, 15784 Athens, Greece

**Keywords:** climate change, GIS analysis, Orchidaceae, orchid distribution, range contraction and expansion, southwards shift, Species Distribution Models (SDMs)

## Abstract

Climate change is regarded as one of the most important threats to plants. Already species around the globe are showing considerable latitudinal and altitudinal shifts. Helen’s bee orchid (*Ophrys helenae*), a Balkan endemic with a distribution center in northwestern Greece, is reported to be expanding east and southwards. Since this southeastern movement goes against the usual expectations, we investigated via Species Distribution Modelling, whether this pattern is consistent with projections based on the species’ response to climate change. We predicted the species’ future distribution based on three different climate models in two climate scenarios. We also explored the species’ potential distribution during the Last Interglacial and the Last Glacial Maximum. *O. helenae* is projected to shift mainly southeast and experience considerable area changes. The species is expected to become extinct in the core of its current distribution, but to establish a strong presence in the mid- and high-altitude areas of the Central Peloponnese, a region that could have provided shelter in previous climatic extremes.

## 1. Introduction

The orchid family is among the earliest divergent angiosperms [1,2] and as such, have a nearly cosmopolitan distribution [3] and comprise more than 28,000 species [4], thus being one of the largest and most diverse plant families in the world. Known for their astonishing morphologies, the complexity of their life cycles, and their high levels of speciation [5,6,7], orchids have always been in the spotlight of scientists, being characterized as a great model for ecological studies to target conservation priorities [8]. Like other plants, orchids are expected to be responding to climate change (cf. [8,9,10,11,12,13]). However, for a number of reasons, such as their reproduction complexity and their dependence on other organisms during their life history, orchids are thought to be more vulnerable [13,14]. 

The last few decades, the trend of global warming is more evident, and the effects on living organisms can be clearly seen [15,16,17,18], with changes in the phenology and distribution of species, as well as possible extinctions attributed to the rapidly changing climate [15,16,19,20]. Many studies have focused on the range changes of species, demonstrating a poleward movement, primarily by the leading edge expanding mainly to the north. Also observed are changes along altitudinal gradients, with species expanding into higher elevations [17,19,21]. Parmesan and Yohe [16] by analyzing ca. 1700 taxa, found that species are facing significant range shifts, with an average poleward movement of 6.1 km per decade. Chen et al. [21] found almost a three times larger shift, with a median rate of a northward shift of 16.9 km per decade, and an uphill shift of 11.0 meters per decade. It is thus clear, that the expected pattern of range changes for plants is a poleward or uphill migration. Although the majority of observations follow this general trend, many have been inconclusive and there is a percentage of species (mainly forest plant taxa) that moved to the opposite direction, southwards and/or downhill, showing a wide variety of range shifts [19,21].

Orchids are no exception when it comes to climate change. Like all plants, Orchidaceae are affected by changes in climatic conditions worldwide, and are reported to follow the general poleward trends and uphill patterns of range shifts. In addition, orchids could face especially significant losses to their distributions, as well as population declines [22,23,24] because of the complexity of their life cycle and their symbiotic dependence on other organisms. In complex symbioses, the responses of all partners to climate change need to be congruous. Thus, any adaptive changes plants make that affect pollination services or the mycorrhizal symbiosis (e.g., [8,22,23]) must be consonant with changes made by their partners. Hence, orchids could face greater risks from global warming [14,25] than other families. This is even more alarming taking into consideration that ca. 40% of all plants are facing greater risk [26] and Orchidaceae are one of the families that are under-assessed regarding their extinction risk status [27]. 

Greece has been extensively floristically explored during the last two centuries [28,29]. Consequently, many studies exist dealing with the factors affecting the biogeographical and biodiversity patterns in Greece ([30] and references therein). Nevertheless, very few studies have taken into consideration the impacts of climate change on plant distribution patterns [31,32,33,34,35] and none has yet assessed if the projected future distribution shifts constitute a physiological response previously experienced by the given taxon in question. After all, incorporating past climate projections in ecological niche modelling may further our understanding of species responses to climate change [36], and could lead to the development of efficient conservation schemes [37].

Being a Mediterranean country with a high topographical and environmental heterogeneity, Greece stands out for its species-rich flora (> ca. 7,500 taxa, 20.4% endemic; [38,39]). One of the most notable aspects of the Greek floral diversity is its orchid species richness (193 orchid species and subspecies; [40]). Orchids can be found nearly everywhere in Greece; 85.4% of the country’s surface hosts orchid species, that are spread across different habitat types, from phrygana and thermophilous pine forests (e.g., several *Anacamptis, Ophrys*, *Serapias* taxa occur in these habitats), to fir forests and alpine grasslands (e.g., *Cephalanthera, Dactylorhiza, Epipactis*) [41]. Most of the orchid species that occur in Greece belong to the genus *Ophrys* (bee orchids, 91 taxa according to [40]), which has its distribution and speciation center in the eastern Mediterranean [40,42]. *Ophrys* is an ideal example of plant-pollinator coevolution, as the majority of *Ophrys* species use sexual deception as a pollination mechanism. By encompassing visual and olfactory signals, they deceive naïve male Hymenoptera to pseudocopulation, and through this procedure, they achieve pollination, and thus, reproduction [43]. Helen’s bee orchid (*Ophrys helenae* Renz) is the only known exception to this rule for *Ophrys*. This Balkan endemic relies not on sexual deception for pollination, but on shelter mimicry [43,44]. 

Recently, Tsiftsis and Tsiripidis [45] assessed the patterns of Greek orchid distributions in time and space, and found that the majority of them, has shown a range increase. Among others, *Ophrys helenae* seems to follow this pattern (see Table 3 in [45]). Described in 1928 on Corfu Island by Dr. Jany Renz [46], its known distribution until the 1980s was restricted to Epirus (NW Greece), and Corfu [47,48]. However, the last few decades, its distribution appears to be expanding, with populations being found in east and southern Greece [40,45]. This observation is intriguing, which seems to run counter to the patterns that are seen due to climate change. Parmesan and Hanley [17] have noted that it is these exceptions to rules have often been the main drivers of advance in ecological understanding.

The purpose of this paper is to investigate whether the conjecture, expressed in [40] for *O. helenae*, that “*…its distribution seems to be undergoing an active expansion towards the east and southern Greece…*”, is consistent with a more systematic analysis, and how climate change will affect the species’ range in the upcoming decades. By using Species Distribution Models (SDMs), we show that this initially puzzling behavior is in fact what we would expect. Moreover, we show that the southward movement of *O. helenae* materializes through an interplay of climatic change with other factors (e.g., topography, soil characteristics, bioclimatic variables, land-cover), and that the species has probably experienced this pattern of shift in the past. 

## 2. Results

The ensemble of small models (ESM) framework predictions was very good, with sufficient predictive power (TSS ≥ 0.95 for all algorithms and the ensemble prediction for both thinning procedures and climate databases, Figure S1). The intrathinning procedure and -climate database variation were statistically insignificant (Kruskal-Wallis ANOVA: H = 7.21, d.f. = 3, *p* = 0.06). 

Precipitation of the wettest month (PWM) had the highest contribution among the response variables, followed by the potential evapotranspiration of the wettest quarter (PET_WQ_) for almost all thinning procedures and climate databases. The only exception was the CHELSA – Geographical Thinning combination (CH_GEO_), where the predicted mean value of soil organic carbon mass fraction at standard depth of 5 cm (SOC-5) was the second most important variable (Table 1).

**Figure 1:**
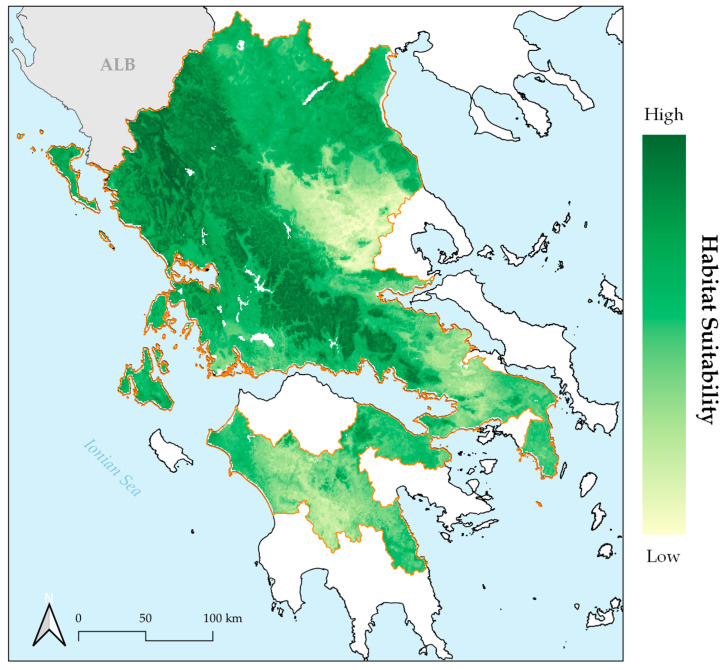
Habitat suitability map for *Ophrys helenae*, for the combination of CHELSA climate database and geographical thinning procedure. Orange border delineates the species’ distributional area based on the Atlas of Greek Orchids [40].

**Figure 2:**
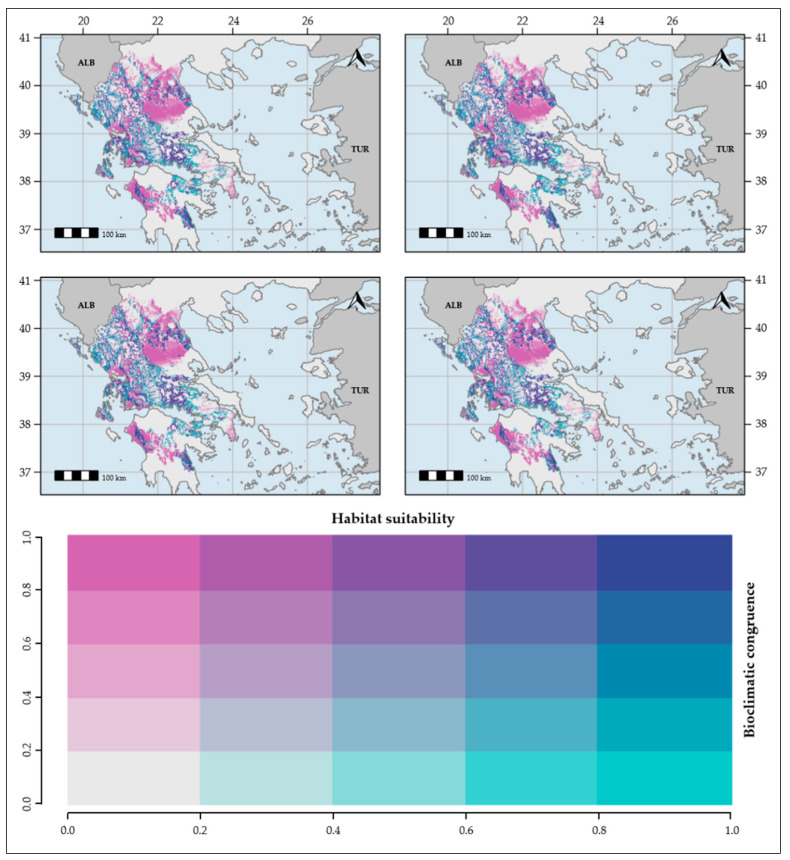
Bioclimatic consistency maps for both thinning procedures (geographical and environmental) of *Ophrys helenae*. From top-left to bottom-right: geographically-thinned, based on WorldClim; geographically-thinned, based on CHELSA; Environmentally-thinned, based on WorldClim; environmentally-thinned, based on CHELSA.

The resulting habitat suitability maps (Figure 1 and Figures S2–S4) had high bioclimatic consistency for every combination of the thinning procedures and distribution areas (Figure 2). They were converted into binary maps, and then compared to the binary maps obtained for each Global Circulation Model (GCM), Representative Concentration Pathway (RCP) scenario, time-period, thinning procedure and climate database. 

**Figure 3:**
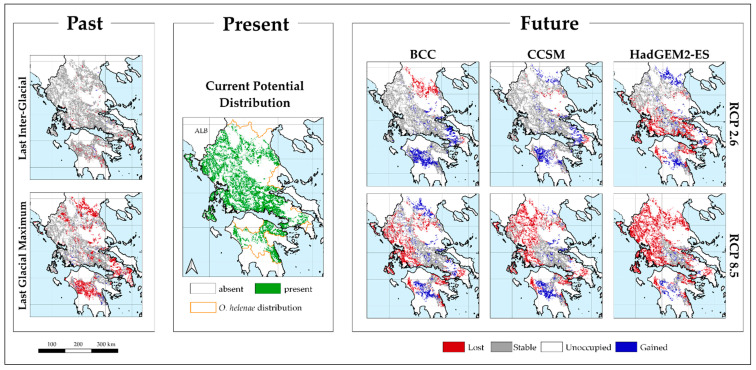
Past, present and future potential distribution maps for *Ophrys helenae*, based on the geographically thinned occurrence data and climate data from CHELSA (CH_GEO_) that show the transition from one time-slice to another. In the central panel, green coloring indicates the cells the species currently potentially occupies, while the orange lines delineate the species’ distributional area. Left-hand panel: past potential distribution maps for showing the transition from the Last Interglacial (ca. 120–140 Kya) to the Last Glacial Maximum Maximum (ca. 20 Kya) and from the Last Glacial Maximum (ca. 20 Kya) to the present time-period. Red, blue and grey coloring depict cells that show loss, gain, or remained stable, respectively, compared to current the previous conditions. Future potential distribution maps represent combinations of three Global Circulation Models (BCC, CCSM4, and HadGEM2-ES) and two Representative Concentration Pathways (RCP 2.6 and RCP 8.5) showing the transition from the present time-period to each respective GCM and RCP combination. The red grid cells in the right-hand panel indicate that the species is currently present at these areas but will not be in the future. The grey grid cells in the right-hand panel indicate that the species is not currently present at these areas and it will not be in the future. The blue grid cells in the right-hand panel indicate that the species currently occupies and will continue to occupy these areas in the future.

As the trends for the future potential distribution of *Ophrys helenae* were largely identical across all uncertainty sources, we selected to present the area-range change for one combination of climatic database and thinning procedure, the CHELSA – Geographical Thinning (CH_GEO_). Our results indicate that the response to climate is expected to contain a number of complexities; *Ophrys helenae* has experienced range expansion and contraction shifts due to periodic climate oscillations (Figure 3 and Figures S5–S7).

**Figure 4:**
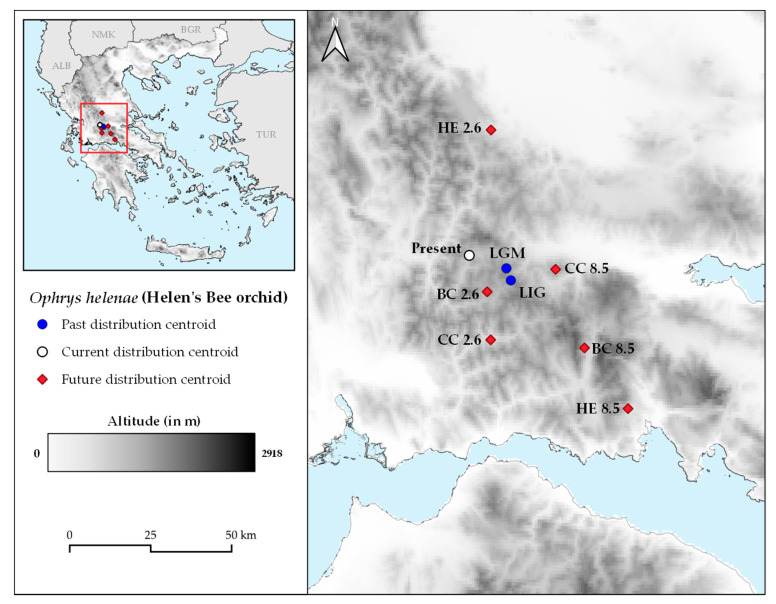
Distribution centroids for current and future projected distributions of *Ophrys helenae* in Greece, based on the combination of CHELSA climatic database and geographical thinning procedure (CH_GEO_) for occurrence data.

The extent of suitable areas attained was largest during the LIG under any thinning procedure/climate database (Figure 3 and Figures S5–S7). Climatically suitable areas for the species underwent slight contraction during the LGM compared to the LIG and moderate contraction in the current period, relative to the LGM. Furthermore, *Ophrys helenae* is expected to face considerable future area changes for all selected GCMs and RCPs, for the presented combination of climatic database and thinning procedure (CH_GEO_: −65.63% to 12.22% depending on the GCM and RCP combination; Table 2 and Figure 3) and all other combinations (Table S1 and Figures S5–S7). In all cases, there is a significant loss of area (Table 2 and Table S1), especially in NW Greece, near the current centre of its range (Figure 3 and Figures S5–S7). However, the species is also projected to expand its southern limits in the near future, shifting its distribution southwards to the Peloponnese, thus resembling its LIG extent of occurrence (Table 2 and Table S1, Figure 3 and Figures S5–S7). The centroids of all projected future distributions of *Ophrys helenae* appear to be lying to the south and southeast of the current distribution centroid, validating the southward shift of the species for future projections (Figure 4), with the sole exception of the HadGEM2 RCP 2.6 projection. The results are consistent through all combinations of climatic databases and occurrence data thinning procedures (Figures S8–S10). 

We detected a statistically significant altitudinal shift between the different time-slices included in our analysis for all climate databases and thinning procedures (Table 3 and Table S2; Kruskal-Wallis ANOVA: H = 14345, d.f. = 8, p < 0.01). *Ophrys helenae* currently occurs in significantly lower altitudes compared to either its past (i.e., in the LIG and the LGM) or its future distribution, with the HadGEM2 RCP8.5 GCM/RCP combination presenting the highest mean altitude among all GCMs, RCPs and time-slices (mean altitude: 915 m a.s.l.; Table 3 and Table S2).

## 3. Discussion

Climate change is expected to continue and to intensify in the future, with a warming effect in a global scale. Plants have been affected by climatic changes in factors that play a major role in their life cycle (e.g., temperature, precipitation) [15,20], resulting in phenological alterations [18,49], as well as in range shifts. This anticipated warming is expected to drive plant populations to shift their distributions on both latitudinal and altitudinal gradients or even become extinct [50,51,52,53]. Species are moving poleward in both hemispheres, while on mountains the altitudinal ranges of species are expected to shift to greater elevation (cf. [16,17,21]). All of these patterns have been observed in numerous studies (e.g., [22,23,25,28,41,42]). However, there are always exceptions to the rule; for example, although most of the shifts observed have been northwards and uphill, there are few cases where plants followed in the opposite direction (see [19,21]). This has led to an increase in biotic homogenization all over the globe, since many range-restricted species are experiencing range contractions, while widespread and alien species are gaining ground, due to the intensifying effects of both climate and land-use change [54,55,56,57,58,59,60].

Orchids, like other plant species, are expected to be affected by the pressures of the changing climate [14]. A number of papers have been written about climate change and orchids, with the majority of them focusing on the physiological effects of changing climate, especially the relationships of orchids with their symbionts (mycorrhizal fungi and pollinators) [8,22,23,61]. The available literature about shifts of orchid distributions (e.g., [62,63,64,65]) has shown that the majority of studied species will be following the usual patterns of distribution change; decreasing of suitable habitat areas of orchids, and northwards or uphill shifts of distribution. Although orchid species that have been studied so far under the prism of climate change are following a distribution shift to higher latitudes and elevations, contrary migration is not an unusual phenomenon for plants and other organisms. Chen et al. [21] found that despite the significant movement northwards and uphill for the majority of species, there has been some movement in the opposite direction, with 22% of the studied taxa shifting towards the equator in latitude, and 25% moving downhill for elevation. Lenoir et al. [19] also found a significant minority going against the mainstream, with ca. 31% of the studied forest plants migrating downhill in W. Europe. The degree of fidelity to the mainstream trend will depend on a variety of functional traits such as generation time, seed mass [66] or, in the case of orchids especially, patterns of life history and fecundity [67].

We found that the expected movement of Helen’s bee orchid is anadromous based on its latitudinal shift; for most of the climatic models and scenarios that we used, the species is moving against the mainstream pattern, shifting its distribution southwards in the coming decades (Figure 4). On the other hand, the species is projected to move uphill in the future, as its mean altitudinal difference reaches ca. 300 m (Table 3). Thus, *Ophrys helenae* seems to be a cold-adapted species based on its response to climate change [16,19,21], as indicated by its distributional and altitudinal shifts from the LIG up to the current time-period, a trend observed for other range-restricted plant species as well [68]. Its slightly lower mean altitude currently observed is directly attributed to the extreme grazing pressure the species is facing in its core distributional area (i.e., Epirus in NW Greece) [69,70,71], that has probably forced many of its populations to extinction. There is a moderate distribution change variation among the GCMs and RCPs that we included in our analysis, but in all cases movement is projected to be mostly south-eastwards. This is in agreement with the pattern observed in [40], but in contrast to the expected poleward trend of most orchids noted in the review of [22]. A northwards range shift was observed by [72] for the lizard orchid (*Himantoglossum hircinum*) in the UK, as well as from [73] for the lady’s slipper orchid (*Cypripedium calceolus*) in Europe. 

*Ophrys helenae* is predicted to experience moderate to high changes in range (Table 2, Figure 3 and Figure 4; see also Supplementary Material), retreating from its northern and western edges, while expanding mainly in the central Peloponnese. Nevertheless, the extreme changes associated with RCP 8.5 for all GCMs will be unequivocally negative for *O. helenae* distribution in Greece, since at least a 20% overall range contraction is predicted to occur. This aligns with the majority of studies for changes in distribution of orchid species. For instance, *Ophrys argolica* and *O. delphinensis*, two orchids occurring in Greece are projected to face a severe loss of their distribution areas, with the latter projected to become extinct [74]. The same holds true for all endemic Cretan orchids [32], which are expected to face severe mean losses of their distribution areas (min: 73.6% - *Epipactis cretica*; max: 99.3% - *Ophrys omegaifera* subsp. *fleischmannii*). In addition, according to [73], *Cypripedium calceolus* will face a major decline of its niche in the future (30–63% loss) under all studied scenarios, the same as for *Epipactis helleborine*, that is expected to undergo significant losses throughout its whole distribution range, despite its wide extent [62]. On the other hand, in the less pessimistic scenario (RCP 2.6), the overall range of Helen’s bee orchid shows a less contraction and may even expand (ca. 15%, CCSM4 model). Similar results were found by [72] for *Himantoglossum hircinum*, which is expected to gain from changing conditions due to climate change, and significantly widen its distribution in the UK northwards. These findings are also in line with those of [75] for Sardinian orchids, such as *Anacamptis papilionacea* var. *papilionacea, Serapias parviflora*, *Ophrys bombyliflora* and *O. morisii*, that are found to significantly expand their distributions in the future under more optimistic RCPs. 

The patterns of change expected for *O. helenae* are complex and not easily contained within a simple description. Lenoir et al. [19] wrote that “…*climate warming does not only affect species at their range boundaries, but its consequences ripple through the whole range of species.*”, That is, climatic change will affect species throughout their range and not just at the boundaries. The simple model of a linearly shifting plant distribution in response to the climatic signal is simplified, just as the notion of plant distributions thin at their edges and aggregating towards an “abundant center” are also simplified [76]. This is clearly demonstrated here where the species is always projected to be extirpated in its current distribution center, and not just at its boundaries.

In this study, we explored *Ophrys helenae* distributions in the past, for two time slices, the Last Glacial Maximum (LGM) and the Last Interglacial (LIG), in order to investigate whether the projected future changes of *O. helenae* are following a recurring pattern. It is notable that a significant portion of the species’ niche is in the mid- to high-altitude areas of central Peloponnese. Thus, when comparing the projected distributions in the prehistoric past, we see that the expected gains in the future distributions, especially south in the central Peloponnese, are returning Helen’s bee orchid to its former “heartlands”. The species will reappear in areas where it should have been at the time of the Last Interglacial, when the climatic conditions were more or less similar to the ones expected in a warmer future [77,78]. 

## 4. Materials and Methods

### 4.1. Study Species

**Figure 5:**
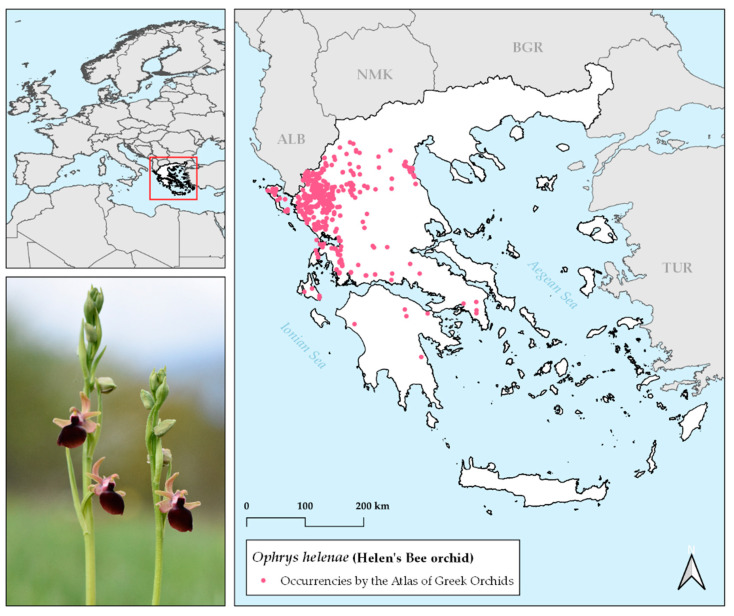
Occurrences of Helen’s bee orchid in Greece, according to the database of the Orchid Flora of Greece project (Atlas of Greek Orchids [40]). Picture of *Ophrys helenae* individuals blooming in Ioannina, NW Greece, April 2019 (photo credits: Kalliopi Stara).

Helen’s bee Orchid (*Ophrys helenae* Renz – sensu [38]) is a Balkan endemic orchid, with its distribution centre lying in northwestern Greece (Figure 5), where it is locally common, while it is also occurring in Albania. A perennial species, with an average height of 26.5 cm, *O. helenae* is one of the most easily recognisable bee orchids, due to its large, cherry-red labellum that lacks a speculum design. In addition, it is the only *Ophrys* species that enlists shelter mimicry as its pollination strategy, rather than sexual deception – the dominant one for bee orchids [43,44]. It can be found in bloom from late March through April and May, in full sun and/or semishade sites among shrubs and forest openings. Recent confirmed observations including populations from East Attica and the Peloponnese reveal an active species’ distribution, with an eastward and southward expansion [40].

### 4.2. Species Occurrence Data

*Ophrys helenae* occurrence data (721 occurrences) were obtained from the database of the Orchid Flora of Greece project ([40] and pers. comm. with Asst. Prof. Spyros Tsiftsis).

We environmentally and geographically thinned the species’ occurrences to avoid pseudoreplication and reduce sampling bias [79,80]. The geographical data cleaning and organizing procedure followed the procedure outlined in [81], using functions from the ‘biogeo’ 1.0 [81] and ‘spThin’ 0.1.0 [80] R packages. The environmental filtering procedure can improve model performance [82] and was based on the representative and uncorrelated environmental variables occurring in the study area (see environmental data below) following [82]. Finally, we evaluated whether any geographical sampling bias existed in our species occurrence data by comparing the statistical distance distribution observed in our dataset to a simulated distribution expected under random sampling via the ‘sampbias’ 1.0.4 [83] R package.

### 4.3. Environmental Data

Current, future and past (Last Glacial Maximum (LGM) and Last Interglacial (LIG)) climatic data were obtained from the WorldClim [84] and the CHELSA [85] databases at a 30 sec resolution (except for LGM data: 2.5 arc-min resolution). We were thus able to assess the bioclimatic consistency and congruence of our models [86] and estimate climate-database uncertainty [87].

We obtained sixteen additional climatic variables at the same resolution via the ‘envirem’ 2.2 [88] R package based on the bioclimatic data from WorldClim and CHELSA for all time-slices. We selected three Global Circulation Models (GCMs) based on [89] and two different Intergovernmental Panel on Climate Change (IPCC) scenarios from the Representative Concentration Pathways (RCP) family: RCP2.6 (mild scenario) and RCP8.5 (severe scenario). We extracted soil variables from the SoilGrids 250 m database [90]. We extracted elevation data from the CGIAR-CSI data-portal [91], which were then aggregated and resampled using functions from the ‘raster’ 3.3.13 R package [92] in order to match the resolution of the other environmental variables. We estimated five topographical variables (slope, aspect, heat load index, topographic position index and terrain ruggedness index) using functions from the ‘raster’ 3.3.13 [92] and ‘spatialEco’ 1.2-0 R packages [93]. We also created a raster layer with the calcareous substrates in Greece, using functions from the ‘sf’ 0.9.6 [94] and the ‘fasterize’ 1.0.3 R package [95], based on the Geological Map of Greece [96].

From this initial set of 50 predictors, only twelve were not highly correlated (Spearman rank correlation < 0.7 and Variance Inflation Factor < 5 – [97]). Multi-collinearity assessment was performed using the ‘vifcor’ function from the ‘usdm’ 1.1.18 [98] R package.

### 4.4. Species Distribution Models

#### 4.4.1. Model Parameterization and Evaluation

We modelled the realized climatic niche of *Ophrys helenae* by combining the available occurrence data with current environmental predictors with the ‘biomod2’ 3.4.6 [99] and ‘ecospat’ 3.1 [100] R packages. We used three different modelling algorithms for our study species: Random Forest (RF), Classification Tree Analysis (CTA) and Artificial Neural Networks (ANN) in an ensemble modelling scheme, to reduce model algorithm uncertainty [87,101]. We generated pseudoabsences following the recommendations of [102] at a minimum distance of 42.9 km from presence locations, which equals the median autocorrelation of the non-collinear environmental variables, using the ‘blockCV’ 2.1.1 [103] R package. We followed the ensemble of small models (ESM) framework [104,105,106], since the occurrence/predictors ratio was lower than 20 [79]. ESMs were subsequently calibrated by fitting bivariate models, which were then averaged into an ensemble model using weights based on model performances. For all models, prevalence was equal to 0.5. We used the True Skill Statistic (TSS; [107]) to evaluate the models’ predictive performance based on a repeated (10 times) 80-20 split-sampling approach. We used null model significance testing [108] to evaluate the performance of our model, which outperformed the null expectation at *p* < 0.001.

#### 4.4.2. Model Projections

We used the calibrated models to predict the suitable area for *Ophrys helenae* under current, future and past conditions [101]. The contribution of each model to the ensemble forecast was weighted according to its TSS score. Models with a TSS score < 0.8 (poorly calibrated models) were excluded from building projections. The final models used for spatial projections were calibrated using 100% of the data. Models were binarised using the value maximizing the TSS score as the threshold for distinguishing presence and absence predictions. As a conservative approach, the suitability of all cells having non-zero values in the clamping mask was set to zero [79]. Finally, we applied a mask representing urban and suburban areas to eliminate any cells that are unsuitable regardless of the environmental conditions.

#### 4.4.3. Area Range Change

Using functions from the ‘biomod2’ 3.4.6 [99] R package, we were able to investigate if *Ophrys helenae* will experience range reduction or increase under future and past conditions. 

### 4.5. Bioclimatic Congruence and Consistency

We followed the framework of [86] in order to construct the bioclimatic congruence and consistency maps for *Ophrys helenae* for every time-period that was available in both climate databases.

### 4.6. Distribution Changes in Latitudinal and Altitudinal Gradient

We estimated the distribution centroids of all cases, in order to test if and how the distribution of *Ophrys helenae* may have shifted in all time-slices (present, past and future), and combinations of climate databases (CHELSA – WorldClim) and occurrence data thinning procedures (Geographical – Environmental), using the ‘st_centroid’ function of the ‘sf’ 0.9.6 [94] R-package. All generated points were plotted in maps via the QGIS v.3.14.16 ‘Pi’ [109]. Also, in order to test the species’ altitudinal shift, we compared the altitude the species is predicted to appear in all time-slices via Kruskal-Wallis tests.

## Figures and Tables

**Table 1:** The two most important variables for each combination of climatic database and thinning procedure. CH_GEO_: CHELSA—Geographical Thinning, CH_ENV_: CHELSA—Environmental Thinning, WC_GEO_: WorldClim—Geographical Thinning, WC_ENV_: WorldClim—Environmental Thinning, PWM: precipitation of wettest month; PET_WQ_: mean monthly potential evapotraspiration of wettest quarter; SOC-5: predicted mean value of soil organic carbon mass fraction at standard depth of 5 cm.

Database/Thinning	Variable Code	Variable Importance
CH_GEO_	PWM	0.874
	SOC-5	0.849
CH_ENV_	PWM	0.899
	PET_WQ_	0.843
WC_GEO_	PWM	0.929
	PET_WQ_	0.855
WC_ENV_	PWM	0.908
	PET_WQ_	0.852

**Table 2:** Percentages of range loss and gain, a overall range change for past and future projections of *Ophrys helenae* distribution. For the past, Last Interglacial and Last Glacial Maximum (LIG and LGM, respectively) are presented, while for the future, all selected Global Circulation Models (GCMs) and Representative Concentration Pathways (RCPs) are presented (BC: BCC-CSM-1, CC: CCSM4, HE: HadGEM2-ES). The presented values are for the CHELSA climatic database, and for the geographical thinning procedure (CH_GEO_).

Database/Thinning	Time Slice	Transition	GCM	Range Loss (%)	Range Gain (%)	Range Change (%)
CH_GEO_	Past	LIG to LGM		4.48	0.67	−3.81
		LGM to Present		29.88	2.14	−27.74
	Future	Present to RCP 2.6	BC	10.43	22.65	12.22
			CC	4.69	19.38	14.68
			HE	35.21	16.97	−18.24
		Present to RCP 8.5	BC	40.04	19.96	−20.08
			CC	47.27	14.37	−32.91
			HE	72.07	6.43	−65.63

**Table 3:** Mean altitude for past, present and future projections of *Ophrys helenae* distribution. For the past, Last Interglacial and Last Glacial Maximum (LIG and LGM, respectively) are presented, while for the future, all selected Global Circulation Models (GCMs) and Representative Concentration Pathways (RCPs). BC: BCC-CSM-1, CC: CCSM4, HE: HadGEM2-ES. The presented values are for the CHELSA climatic database and geographical thinning procedure (CH_GEO_).

Database/Thinning	Time Slice	RCP	Period/GCM	Mean Altitude (m)
CH_GEO_	Past		LIG	671
			LGM	679
	Current		Present	637
	Future	RCP 2.6	BC	653
			CC	634
			HE	866
		RCP 8.5	BC	712
			CC	860
			HE	915

## Data Availability

Not applicable.

## Supplementary Materials

The following are available online at https://www.mdpi.com/2223-7747/10/3/470/s1, Figure S1. True Skill Statistic (TSS) values for every source of uncertainty included in the present study. TH_GEO_: geographical thinning. TH_ENV_: environmental thinning. HU_ALPHA_: distribution area estimated with the alpha-hull method. CH: CHELSA climate database. WC: WorldClim climate database. Panels A, B, and C correspond to the thinning procedure, climate database, and all the aforementioned uncertainty sources combined, respectively. Figure S2. Habitat suitability map for *Ophrys helenae*, for the combination of CHELSA climate database and environmental thinning procedure. Orange border delineates the species’ distributional area based on the Atlas of Greek Orchids. Figure S3. Habitat suitability map for *Ophrys helenae*, for the combination of WorldClim climate database and geographical thinning procedure. Orange border delineates the species’ distributional area based on the Atlas of Greek Orchids. Figure S4. Habitat suitability map for *Ophrys helenae*, for the combination of WorldClim climate database and environmental thinning procedure. Orange border delineates the species’ distributional area based on the Atlas of Greek Orchids. Figure S5. Past, present and future potential distribution maps for *Ophrys helenae*, based on the environmentally thinned occurrence data and climate data from CHELSA (CH_ENV_) that show the transition from one time-slice to another. In the central panel, green coloring indicates the cells the species currently potentially occupies, while the orange lines delineate the species’ distributional area. Left-hand panel: past potential distribution maps for showing the transition from the Last Interglacial (ca. 120–140 Kya) to the Last Glacial Maximum (ca. 20 Kya) and from the Last Glacial Maximum (ca. 20 Kya) to the present time-period. Red, blue and grey coloring depict cells that show loss, gain, or remained stable, respectively, compared to current the previous conditions. Future potential distribution maps represent combinations of three Global Circulation Models (BCC, CCSM4, and HadGEM2-ES) and two Representative Concentration Pathways (RCP 2.6 and RCP 8.5) showing the transition from the present time-period to each respective GCM and RCP combination. The red grid cells in the right-hand panel indicate that the species is currently present at these areas but will not be in the future. The grey grid cells in the right-hand panel indicate that the species is not currently present at these areas and it will not be in the future. The blue grid cells in the right-hand panel indicate that the species currently occupies and will continue to occupy these areas in the future. Figure S6. Past, present and future potential distribution maps for *Ophrys helenae*, based on the geographically thinned occurrence data and climate data from WorldClim (WC_GEO_) that show the transition from one time-slice to another. In the central panel, green coloring indicates the cells the species currently potentially occupies, while the orange lines delineate the species’ distributional area. Left-hand panel: past potential distribution maps for showing the transition from the Last Interglacial (ca. 120–140 Kya) to the Last Glacial Maximum (ca. 20 Kya) and from the Last Glacial Maximum (ca. 20 Kya) to the present time-period. Red, blue and grey coloring depict cells that show loss, gain, or remained stable, respectively, compared to current the previous conditions. Future potential distribution maps represent combinations of three Global Circulation Models (BCC, CCSM4, and HadGEM2-ES) and two Representative Concentration Pathways (RCP 2.6 and RCP 8.5) showing the transition from the present time-period to each respective GCM and RCP combination. The red grid cells in the right-hand panel indicate that the species is currently present at these areas but will not be in the future. The grey grid cells in the right-hand panel indicate that the species is not currently present at these areas and it will not be in the future. The blue grid cells in the right-hand panel indicate that the species currently occupies and will continue to occupy these areas in the future. Figure S7. Past, present and future potential distribution maps for Ophrys helenae, based on the environmentally thinned occurrence data and climate data from WorldClim (WC_ENV_) that show the transition from one time-slice to another. In the central panel, green coloring indicates the cells the species currently potentially occupies, while the orange lines delineate the species’ distributional area. Left-hand panel: past potential distribution maps for showing the transition from the Last Interglacial (ca. 120–140 Kya) to the Last Glacial Maximum (ca. 20 Kya) and from the Last Glacial Maximum (ca. 20 Kya) to the present time-period. Red, blue and grey coloring depict cells that show loss, gain, or remained stable, respectively, compared to current the previous conditions. Future potential distribution maps represent combinations of three Global Circulation Models (BCC, CCSM4, and HadGEM2-ES) and two Representative Concentration Pathways (RCP 2.6 and RCP 8.5) showing the transition from the present time-period to each respective GCM and RCP combination. The red grid cells in the right-hand panel indicate that the species is currently present at these areas but will not be in the future. The grey grid cells in the right-hand panel indicate that the species is not currently present at these areas and it will not be in the future. The blue grid cells in the right-hand panel indicate that the species currently occupies and will continue to occupy these areas in the future. Figure S8. Distribution centroids for past, present and future projected distributions of *Ophrys helenae* in Greece, for the combination of CHELSA climatic database, and environmental thinning procedure for occurrence data. Figure S9. Distribution centroids for past, present and future projected distributions of *Ophrys helenae* in Greece, for the combination of WorldClim climatic database, and geographical thinning procedure for occurrence data. Figure S10. Distribution centroids for past, present and future projected distributions of *Ophrys helenae* in Greece, for the combination of WorldClim climatic database, and environmental thinning procedure for occurrence data. Table S1. Percentages of range loss and gain, an overall range change for past and future projections of *Ophrys helenae* distribution. For the past, Last Interglacial and Last Glacial Maximum (LIG and LGM, respectively) are presented, while for the future, all selected Global Circulation Models (GCMs) and Representative Concentration Pathways (RCPs). BC: BCC-CSM-1, CC: CCSM4, HE: HadGEM2-ES. The presented values are for all climatic databases, and occurrence data thinning procedures. Table S2. Mean altitude for past, present and future projections of *Ophrys helenae* distribution. For the past, Last Interglacial and Last Glacial Maximum (LIG and LGM, respectively) are presented, while for the future, all selected Global Circulation Models (GCMs) and Representative Concentration Pathways (RCPs). BC: BCC-CSM-1, CC: CCSM4, HE: HadGEM2-ES. The presented values are for all climatic databases, and occurrence data thinning procedures.
