# Supplementary figures and images for: An Orchid in Retrograde: Climate-Driven Range Shift Patterns of Ophrys helenae in Greece
Source: Plants (Basel). 2021 Mar 2;10(3):470. doi: 10.3390/plants10030470 (PMC8000551; doi:10.3390/plants10030470)

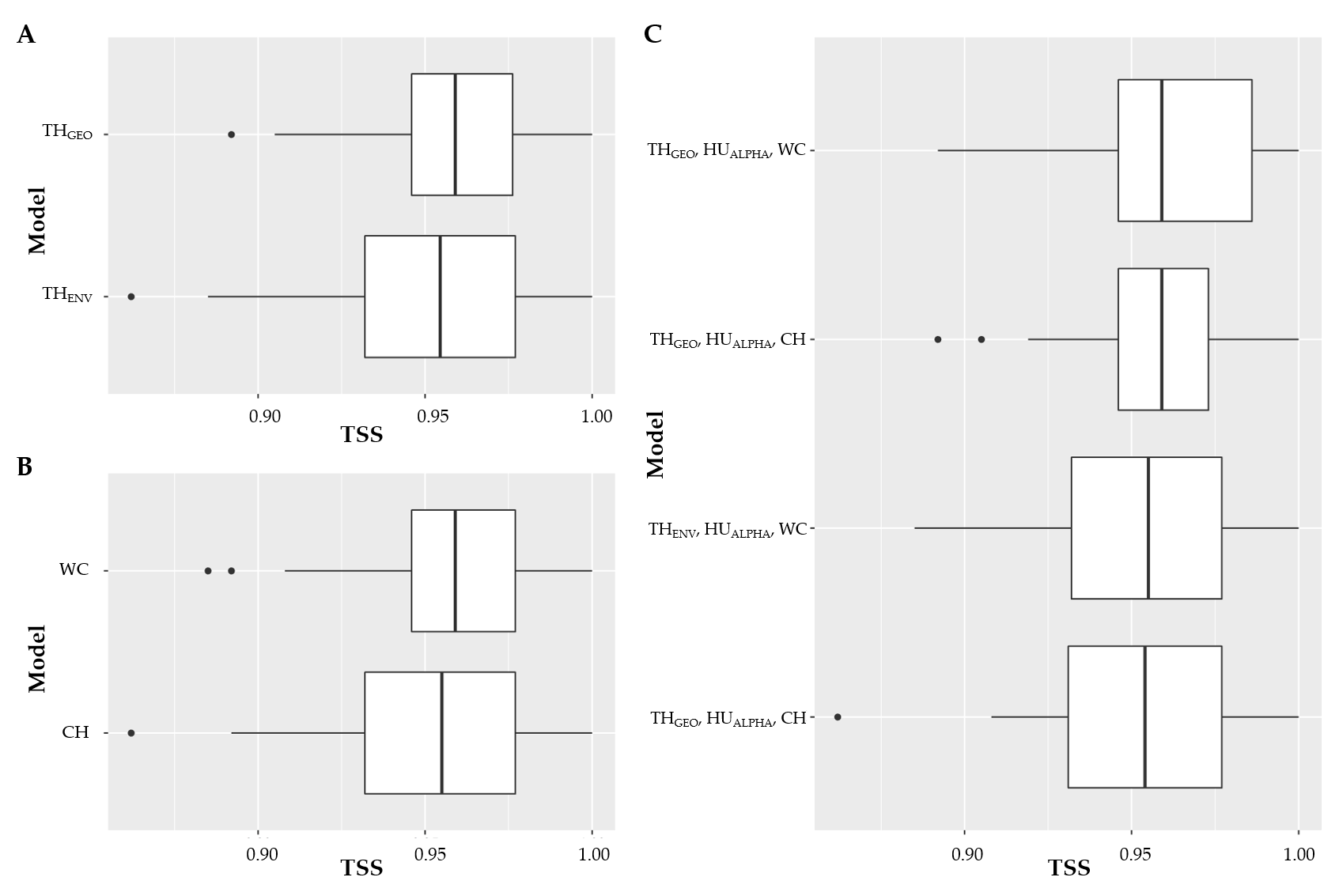

Supplement: Supplementary file 1 [file plants-10-00470-s001.zip › Figure S1.png]

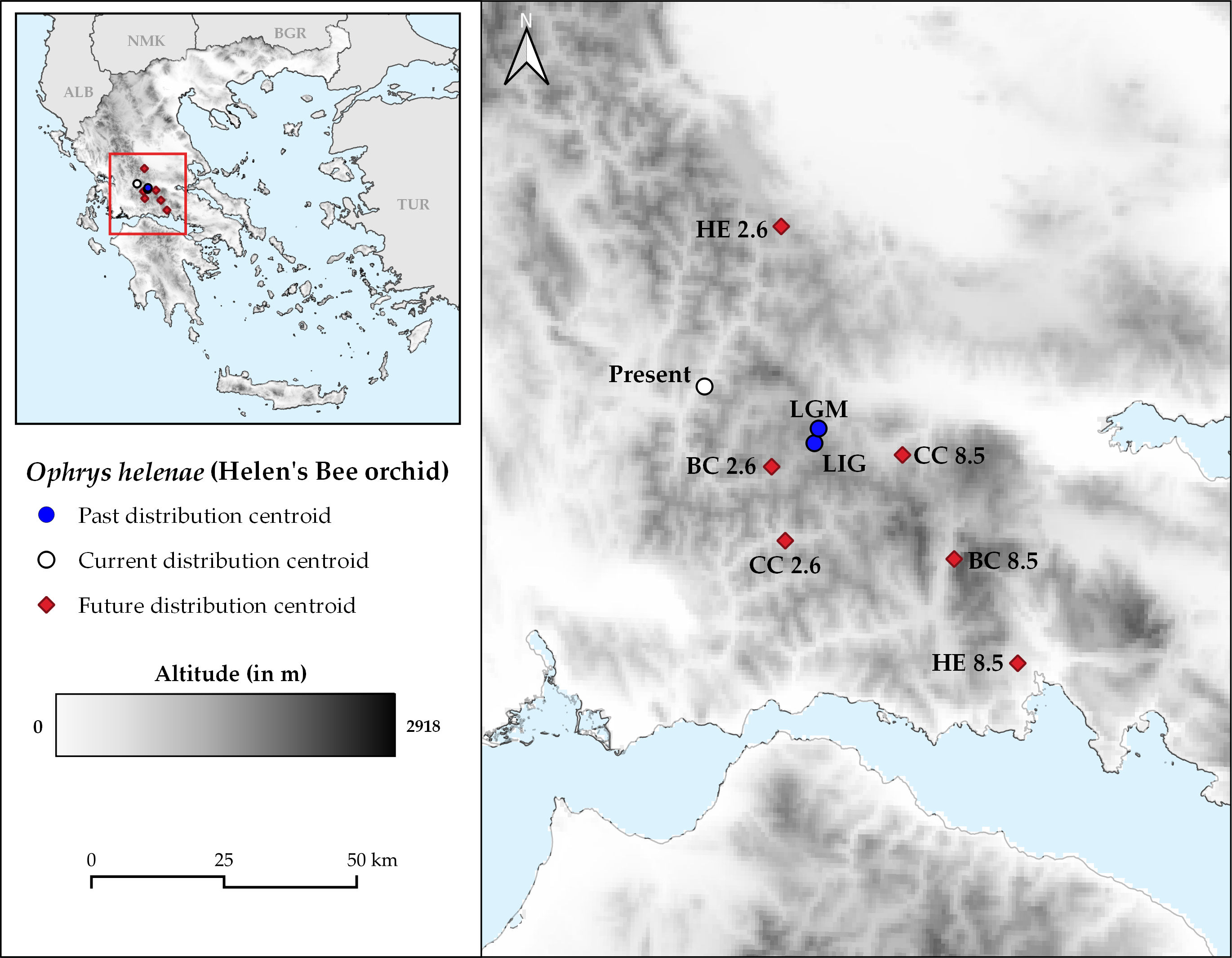

Supplement: Supplementary file 1 [file plants-10-00470-s001.zip › Figure S10.png]

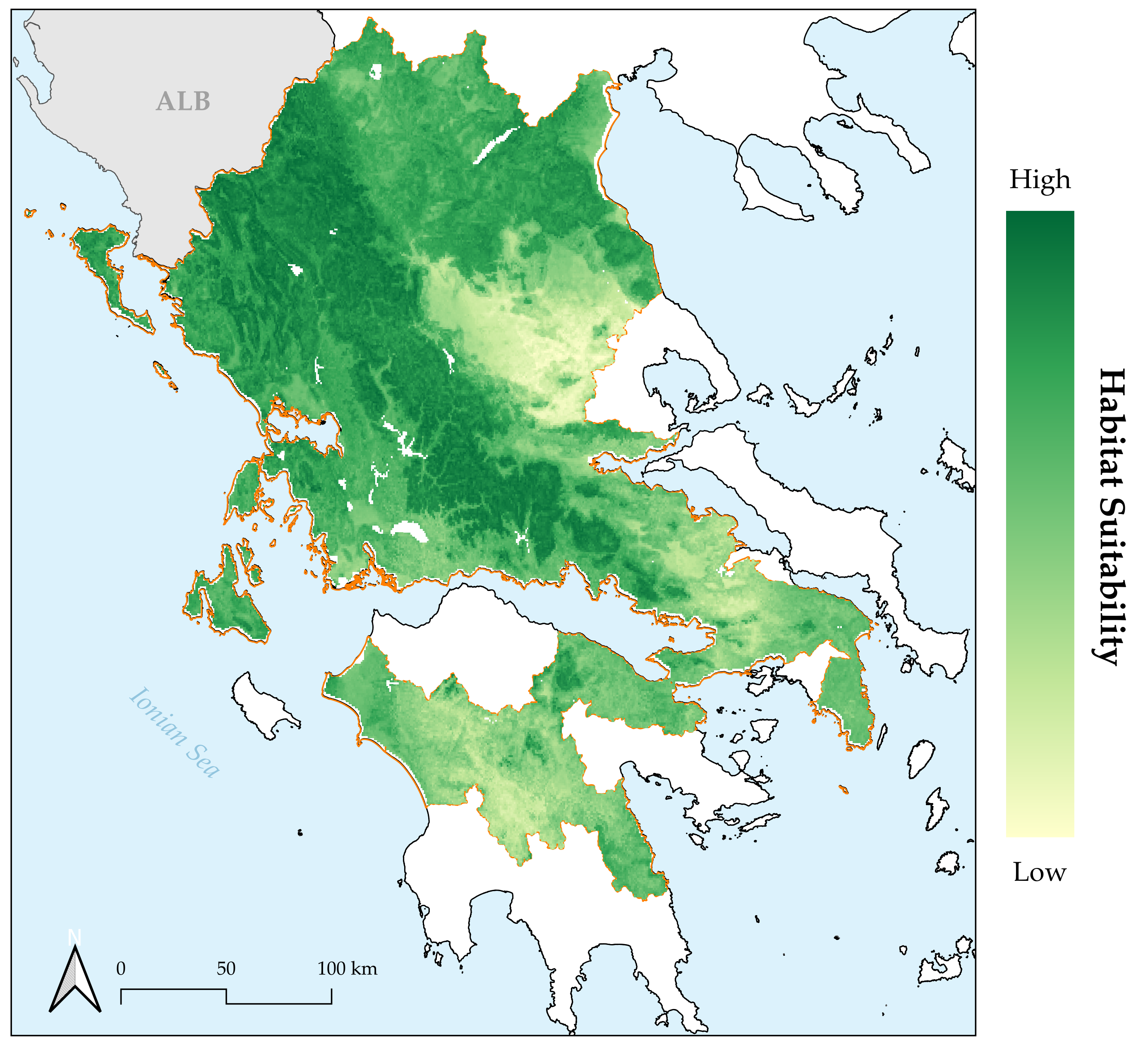

Supplement: Supplementary file 1 [file plants-10-00470-s001.zip › Figure S2.png]

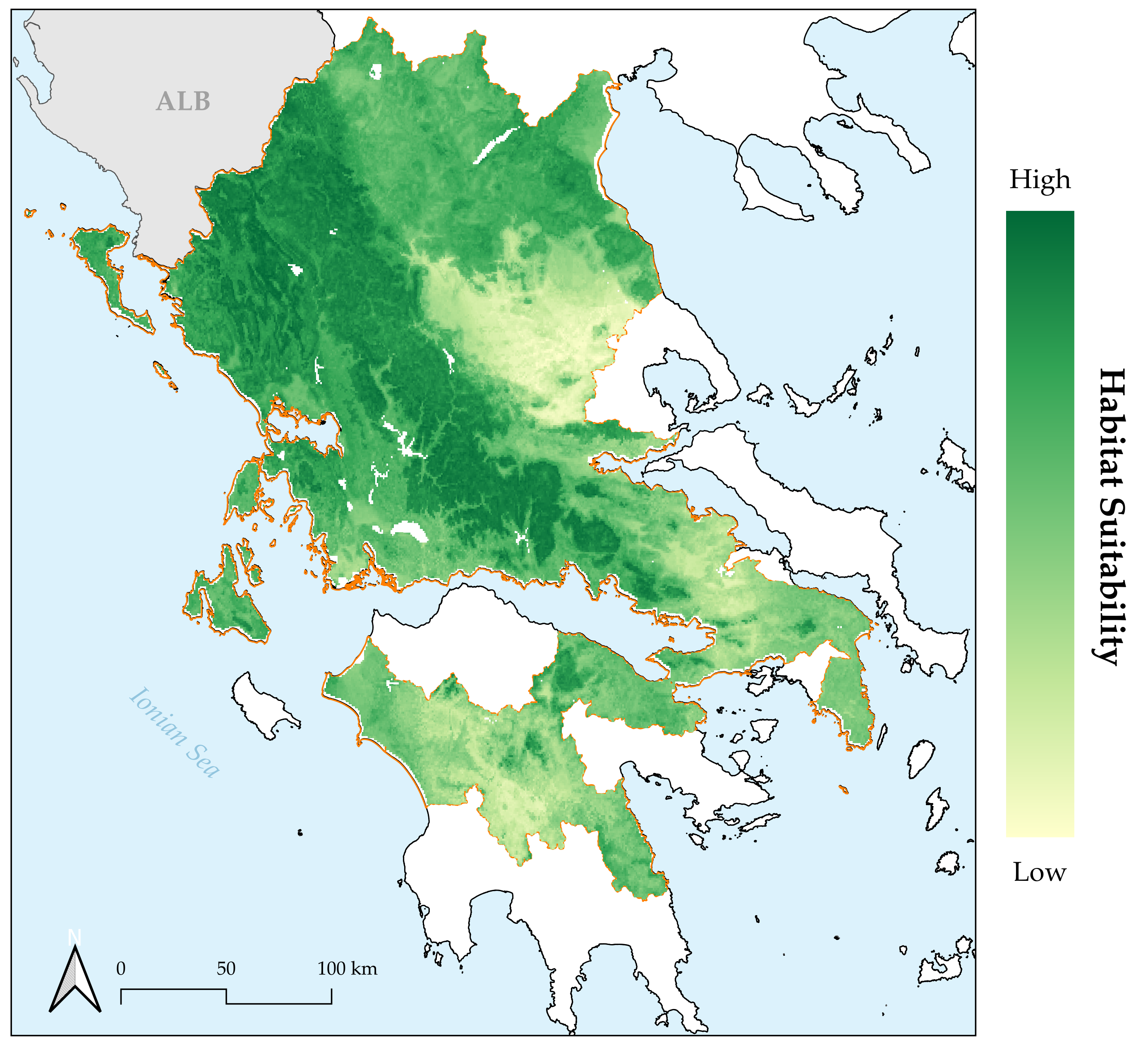

Supplement: Supplementary file 1 [file plants-10-00470-s001.zip › Figure S3.png]

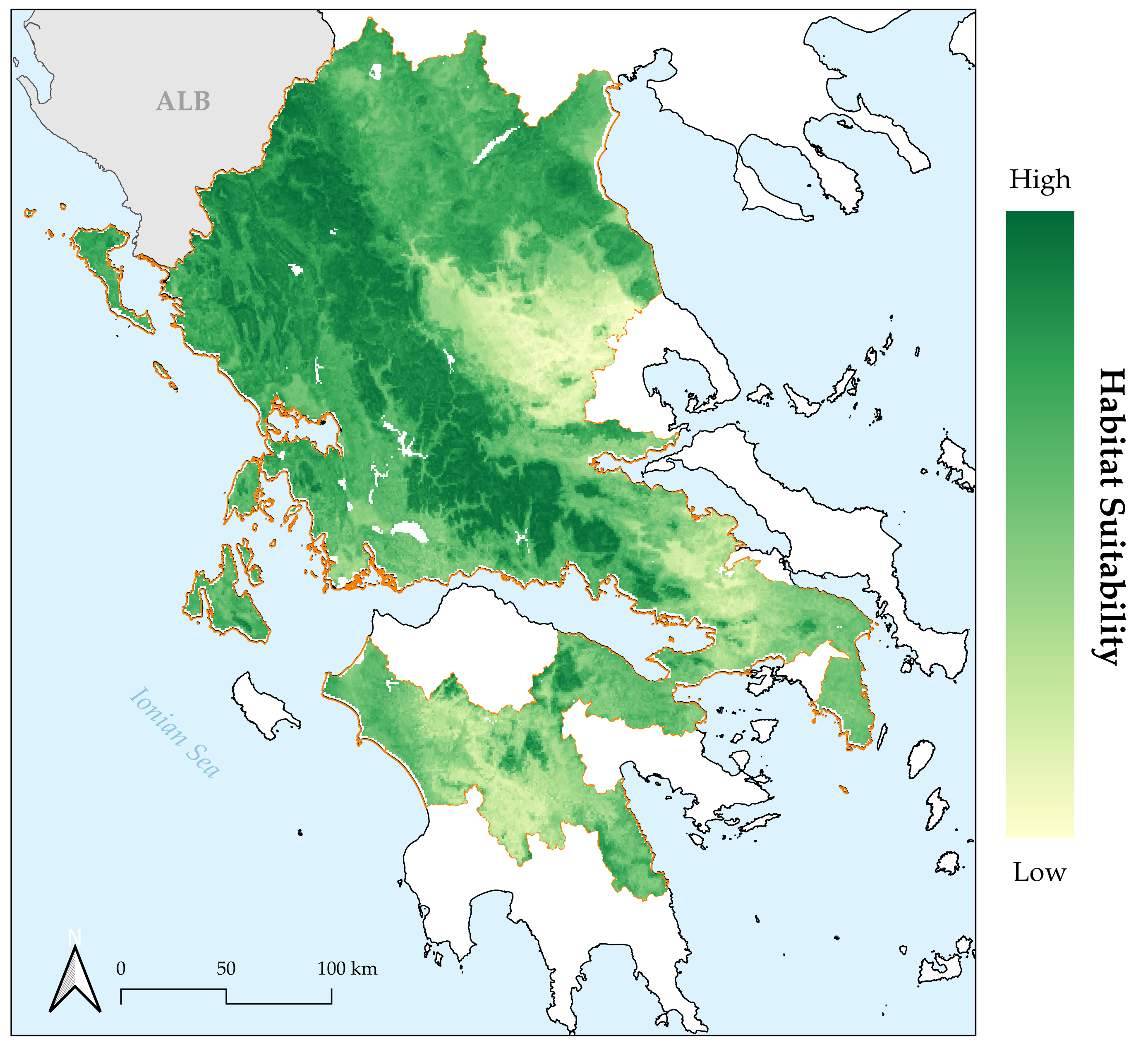

Supplement: Supplementary file 1 [file plants-10-00470-s001.zip › Figure S4.png]

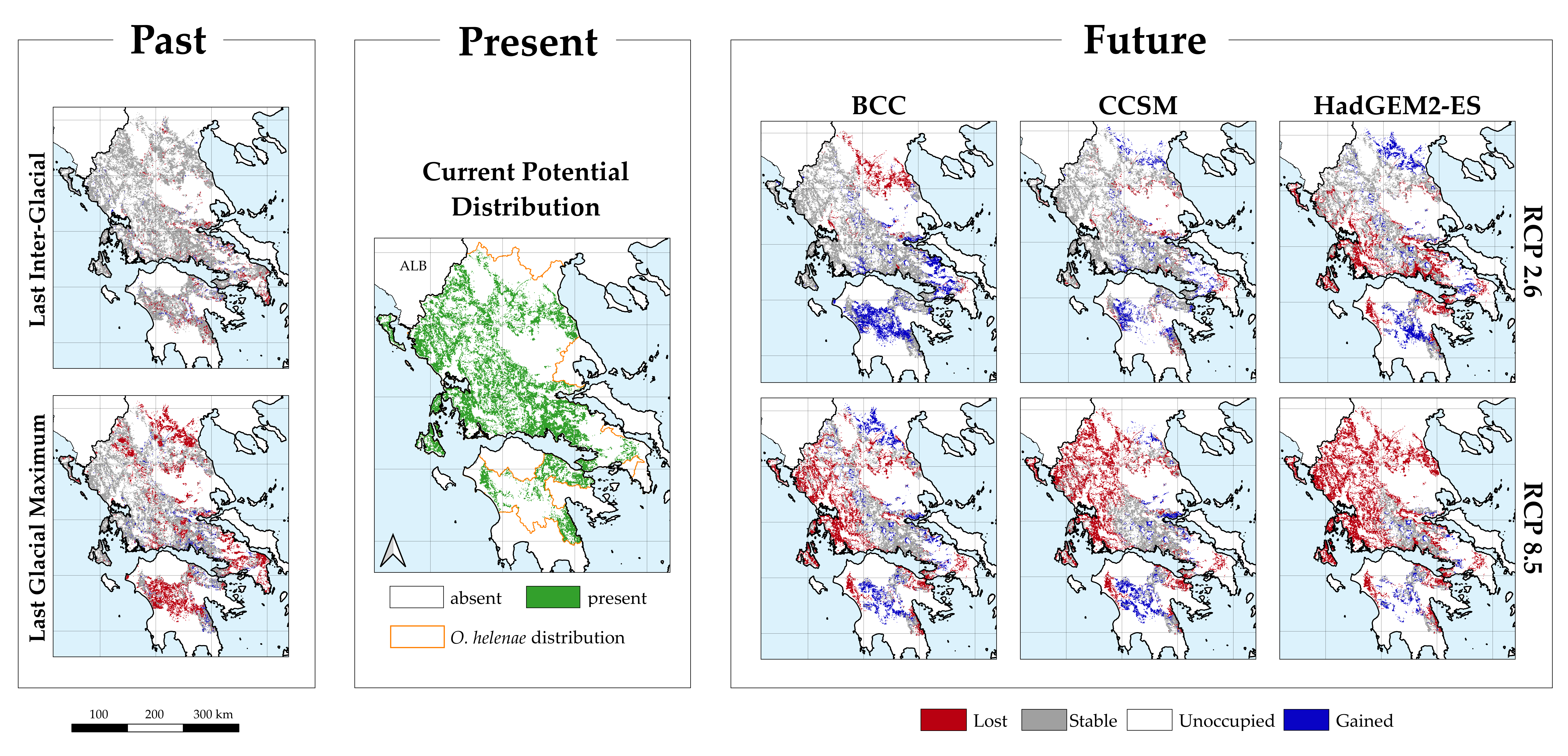

Supplement: Supplementary file 1 [file plants-10-00470-s001.zip › Figure S5.tiff]

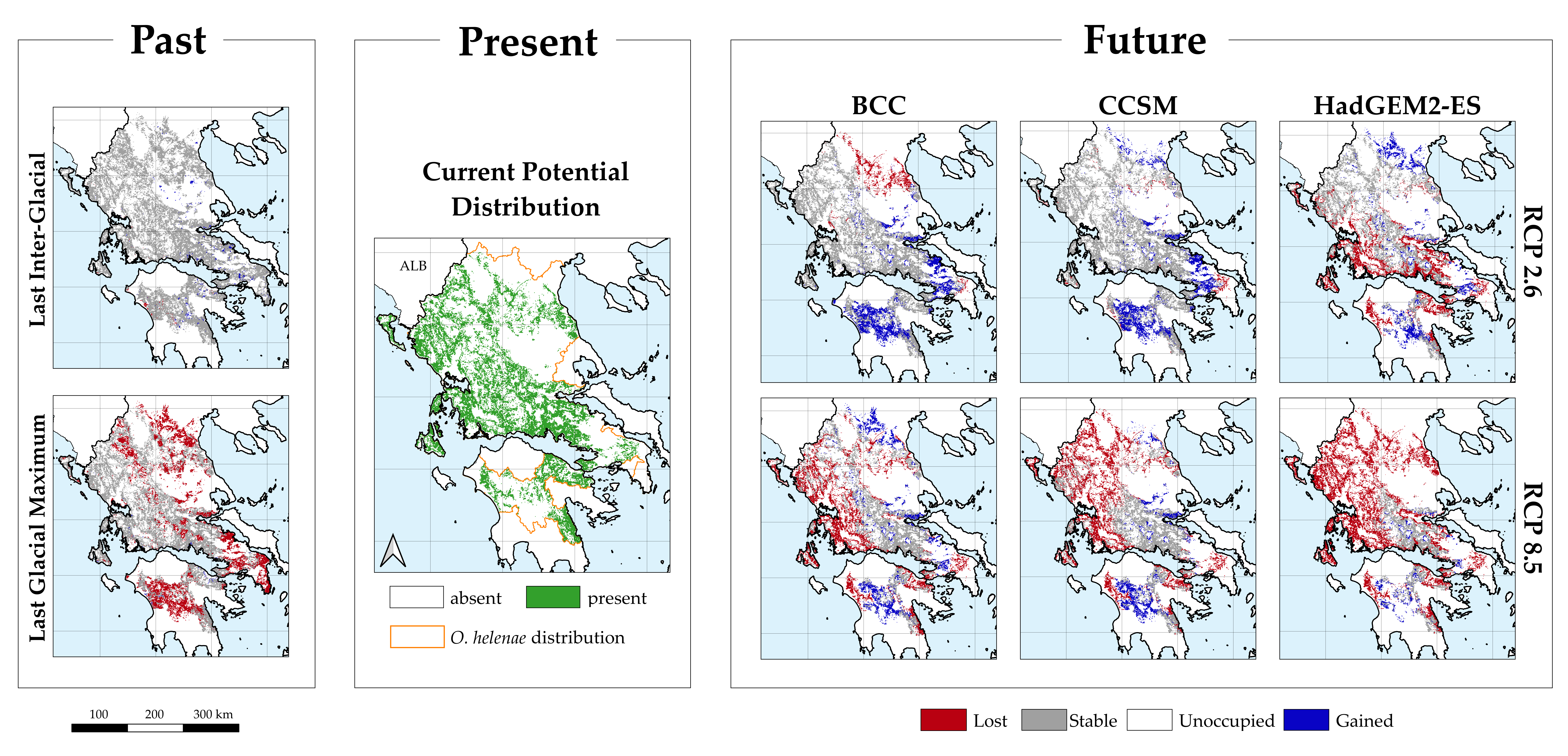

Supplement: Supplementary file 1 [file plants-10-00470-s001.zip › Figure S6.tiff]

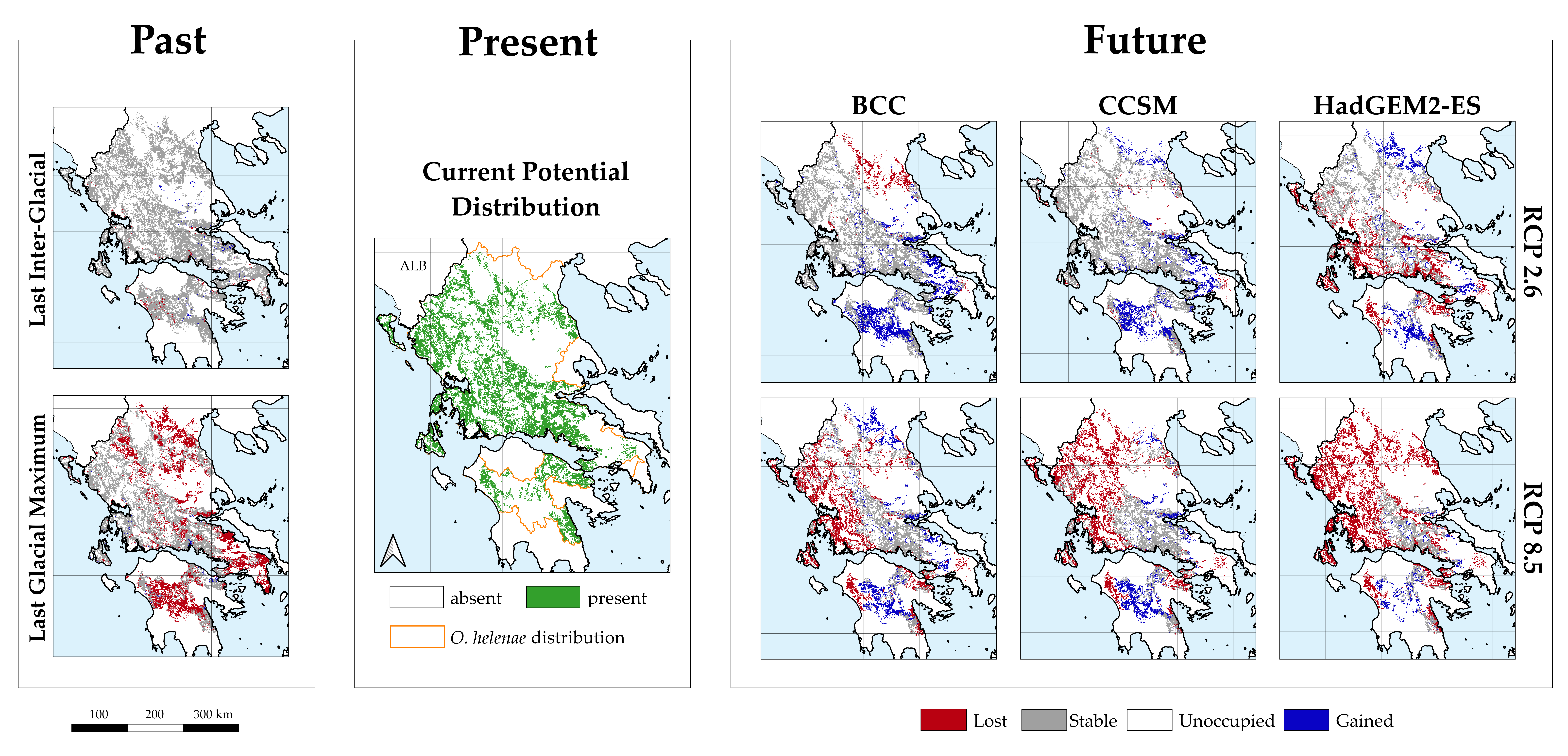

Supplement: Supplementary file 1 [file plants-10-00470-s001.zip › Figure S7.tiff]

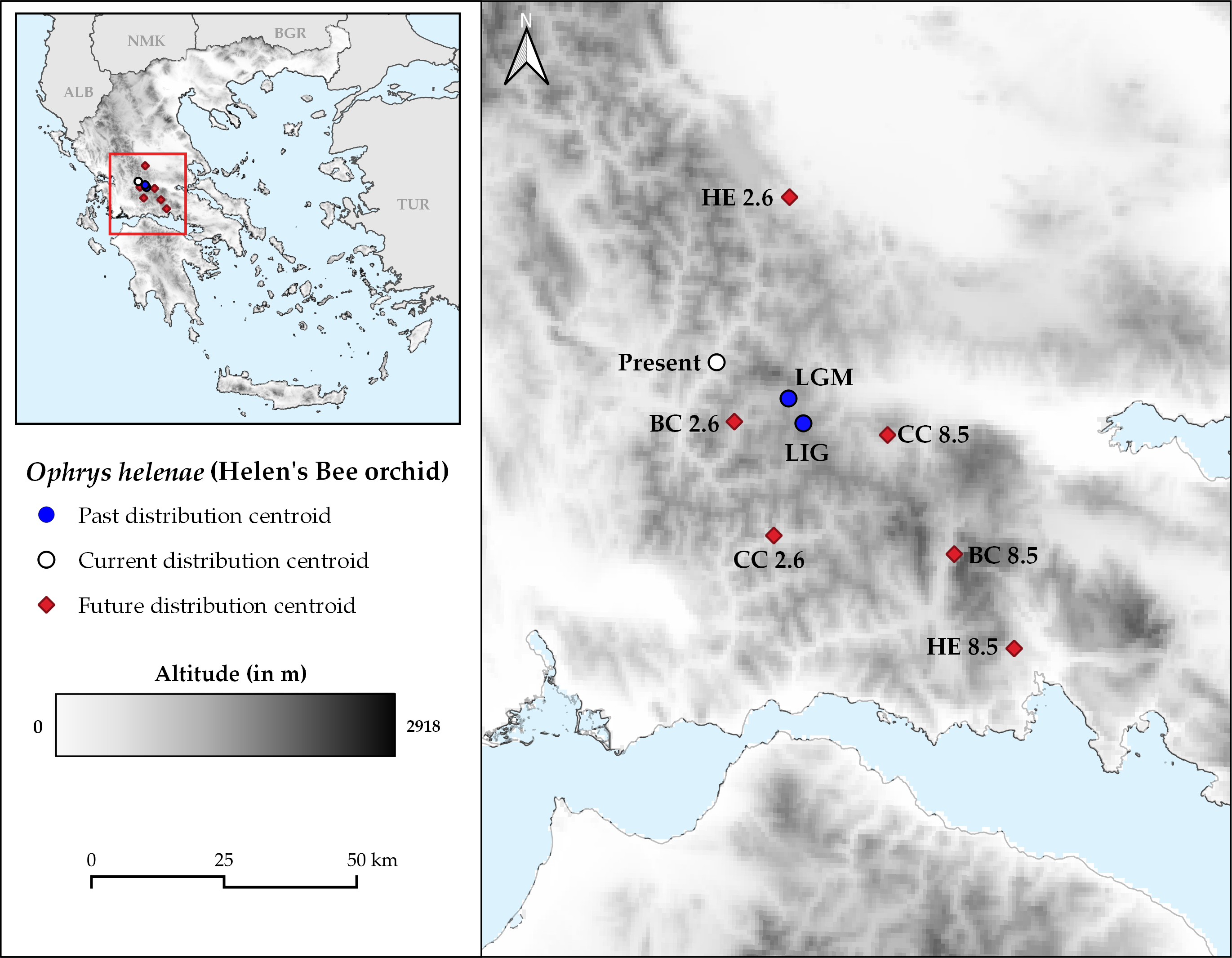

Supplement: Supplementary file 1 [file plants-10-00470-s001.zip › Figure S8.png]

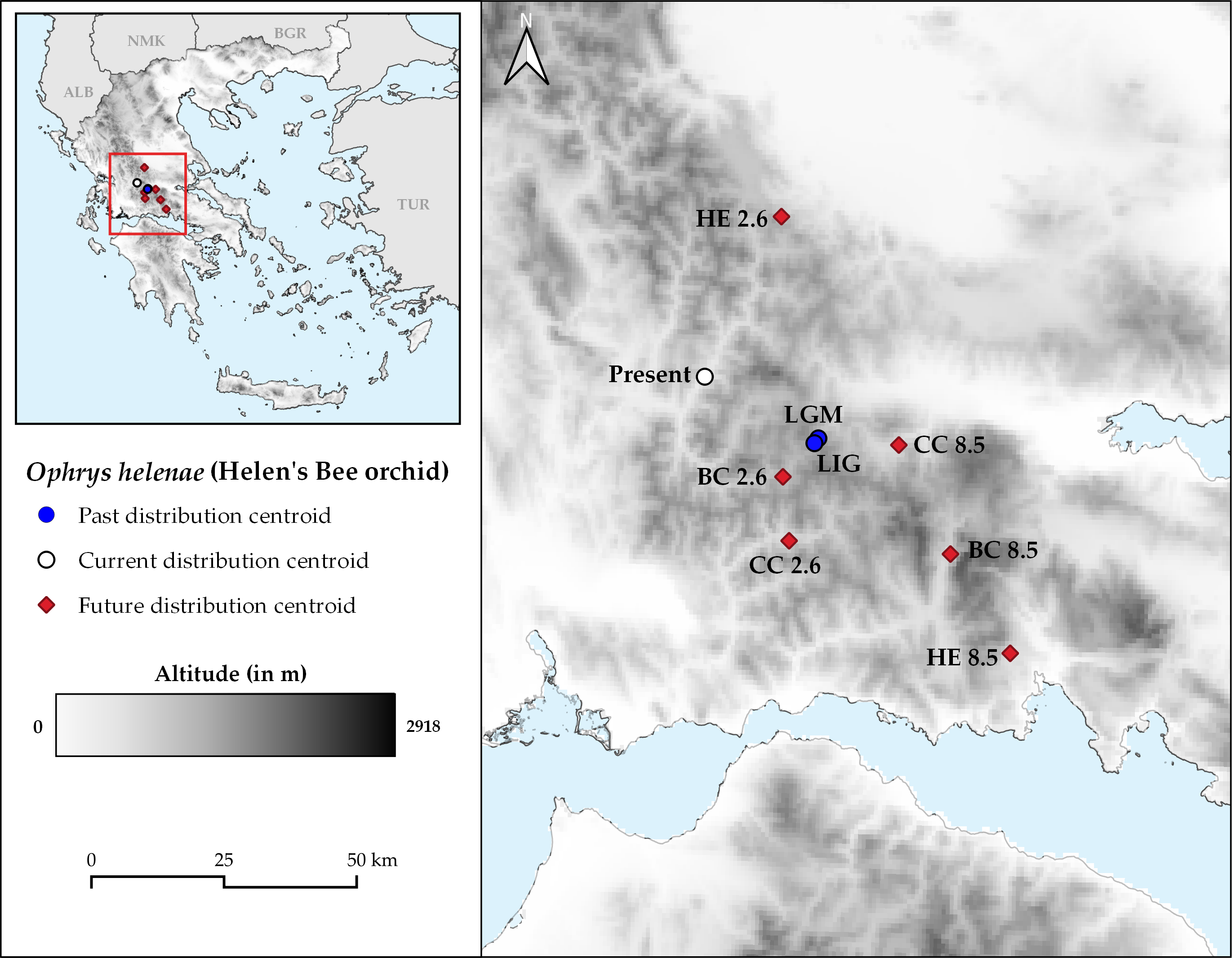

Supplement: Supplementary file 1 [file plants-10-00470-s001.zip › Figure S9.png]
